# Romosozumab in postmenopausal women with smoldering multiple myeloma: a prospective 12-mo study

**DOI:** 10.1093/jbmrpl/ziaf144

**Published:** 2025-08-30

**Authors:** Mariana Diz Lopes, Francesco Pollastri, Francesca Mastropaolo, Rosanna Somma, Mattia Tugnolli, Emma Pasetto, Camilla Benini, Valeria Messina, Davide Gatti, Ombretta Viapiana, Maurizio Rossini, Elena Marchetti, Martina Tinelli, Giovanni Adami

**Affiliations:** Rheumatology Section, Department of Medicine, University of Verona, Verona 37134, Italy; Rheumatology Department, Unidade Local de Saúde de São João, Porto 4200-319 Portugal; Rheumatology Section, Department of Medicine, University of Verona, Verona 37134, Italy; Rheumatology Section, Department of Medicine, University of Verona, Verona 37134, Italy; Rheumatology Section, Department of Medicine, University of Verona, Verona 37134, Italy; Rheumatology Section, Department of Medicine, University of Verona, Verona 37134, Italy; Rheumatology Section, Department of Medicine, University of Verona, Verona 37134, Italy; Rheumatology Section, Department of Medicine, University of Verona, Verona 37134, Italy; Rheumatology Section, Department of Medicine, University of Verona, Verona 37134, Italy; Rheumatology Section, Department of Medicine, University of Verona, Verona 37134, Italy; Rheumatology Section, Department of Medicine, University of Verona, Verona 37134, Italy; Rheumatology Section, Department of Medicine, University of Verona, Verona 37134, Italy; Hematology Section, Department of Medicine, University of Verona, Verona 37134, Italy; Hematology Section, Department of Medicine, University of Verona, Verona 37134, Italy; Rheumatology Section, Department of Medicine, University of Verona, Verona 37134, Italy

**Keywords:** multiple myeloma, osteoporosis, romosozumab, BMD, bone turnover markers

## Abstract

Bone damage occurs early in the progression of plasma cell disorders. Romosozumab (ROMO), a sclerostin inhibitor with both anabolic and antiresorptive effects, may provide therapeutic benefit for this fracture-prone population. We conducted a 12-mo prospective observational study on postmenopausal women with osteoporosis and multiple myeloma (MM) treated with monthly ROMO. BMD was assessed at baseline, 6, and 12 mo with DXA and HR-pQCT. Additionally, bone turnover markers (BTMs) and several MM markers were also monitored. Eight female patients with MM without evidence of CRAB completed 12 mo of ROMO. A significant BMD increase was observed at the LS (+5.8%, *p* = .048), FN (+4.2%, *p* = .020), and TH (+3.3%, *p* = .002). Procollagen I intact N-terminal peptide rose sharply at month 3 (+117.8%) and returned to baseline by month 12 (*p* = .006). C-terminal telopeptide of type I collagen decreased progressively, showing a trend toward significance (−53.6%, *p* = .060). Alkaline phosphatase and bone alkaline phosphatase significantly declined by month 12 (*p* = .020 and *p* = .022). No significant changes occurred in immunoglobulins, M-protein, or light chains. β2-microglobulin decreased at month 12 (from 2.35 to 2.1 mg/L, *p* = .042), but the overall trend was not significant (*p* = .092). Failure load increased at month 6 (*p* = .033), while the other HR-pQCT parameters remained stable throughout the study. In this exploratory study of postmenopausal women with MM and osteoporosis, ROMO significantly improved BMD and modulated BTMs, without any evidence of disease progression over the 12-mo period. These findings support ROMO as a potential bone-targeted therapy in patients with osteoporosis and MM.

## Introduction

Multiple myeloma (MM) is a severe hematological malignancy characterized by uncontrolled proliferation of plasma cells (PCs) within the bone marrow (BM).^1^ Multiple myeloma-associated bone disease is characterized by increased bone resorption, suppressed osteoblast (OB) function, and associated lytic bone lesions.^2^ Multiple myeloma-associated bone disease is also characterized by a predominant impairment of bone formation, highlighting the need to protect and rebuild the structural integrity of bone.^3^ Multiple myeloma-associated bone disease increases the risk of skeletal-related events (SREs), which are defined as pathological fractures, spinal cord compression, or the need for surgical or radiotherapeutic intervention.^4^

Multiple myeloma diagnosis according to the revised International Myeloma Working Group (IMWG) criteria requires the presence of at least one of myeloma defining events (MDE) in addition to the accumulation of >10% clonal BM PCs.^5^ Myeloma defining events include the established CRAB (hypercalcemia, renal failure, anemia, or bone-disease related osteolytic lesions) but also clonal BM PCs ≥60%, serum free light chain (FLC) ratio ≥100 or more than one focal lesion on magnetic resonance imaging. Emerging evidence demonstrated that bone damage begins early in the course of MM with BMD loss, microarchitectural defects, and higher fracture risk.^6–9^

In MM, bone homeostasis is severely disrupted.^10^^,^^11^ Myeloma PCs interact with OBs, osteoclasts (OCs), and BM stromal cells, releasing signaling molecules that promote bone destruction.^2^^,^^10–12^ Osteoclast activation in MM is driven by multiple pathways, including the RANK/RANKL axis, Notch signaling, and other osteoclastogenic factors.^10^^,^^13^^,^^14^ Meanwhile, OB suppression is largely attributed to dysregulated Wnt signaling, primarily through elevated levels of its inhibitors, Dickkopf-1 (DKK1) and sclerostin (SOST).^15–19^ Multiple myeloma cells also induce osteocyte apoptosis, which further amplifies SOST release, enhancing OC activity, and suppressing bone formation.^18^^,^^20^^,^^21^ Importantly, apoptotic osteocytes contribute to remodeling the BM niche, promoting MM cell homing and expansion.^21^^,^^22^

Serum SOST levels are elevated in MM and correlate with reduced osteoblast function, disease stage, and fracture risk.^23^ Targeting SOST has therefore emerged as a promising therapeutic strategy.^24^ In MM mouse models, anti-SOST antibodies restored bone formation and reversed bone lesions.^25^

Despite advancements in MM treatment, current bone-targeted therapies for SREs are limited to bisphosphonates and denosumab.^26^ Romosozumab (ROMO), a monoclonal antibody against SOST, stimulates bone formation while inhibiting resorption. Approved for treatment of severe osteoporosis in postmenopausal women at high risk of fracture, ROMO significantly increases BMD and reduces fracture incidence.^27^^,^^28^ Given its dual mechanism, ROMO may offer added benefit in MM by improving bone health and potentially altering the tumor-supportive microenvironment, particularly in early-stage.

In this exploratory study in patients with MM and osteoporosis, we aimed to evaluate the impact of ROMO in bone health and in MM disease activity.

## Materials and methods

### Study design

We conducted a 12-mo prospective observational study on patients with MM and severe osteoporosis who received ROMO 210 mg/monthly for 12 mo between October 2023 and April 2025. All patients received vitamin D supplementation (cholecalciferol 1000 UI/day) throughout the study.

### Inclusion criteria

- Diagnosis of MM according to the revised IMWG criteria without evidence of CRAB and not being treated for MM.
- Treatment with ROMO as deemed necessary by the treating physician.

### Exclusion criteria

- History of myocardial infarction or stroke.
- Bone diseases other than osteoporosis (eg, Paget’s disease or osteomalacia).
- History of bone malignancy.
- Severe liver or kidney disease (eGFR <30 mL/min or Child–Pugh grade B/C).
- Uncontrolled endocrine disorders (eg, hypocalcemia, primary hyperparathyroidism).
- Naïve to bisphosphonates and denosumab either for myeloma treatment or osteoporosis and to any other anti-osteoporosis treatment.

Our primary outcome was the changes in LS BMD at 12 mo of treatment with ROMO. We additionally evaluated changes in BMD in other sites, bone turnover markers (BTMs), MM activity markers, and HR-pQCT over the follow-up time.

### Data collection

BMD was measured at baseline, month 6, and month 12, at the FN, TH, and LS (L1-L4) using DXA with the Lunar iDXA machine (the variation coefficient for the vertebral site was 1%, while it was 1.2% for the FN). Vertebral fracture assessment (VFA) was performed to assess for new vertebral fractures at follow-up timepoints.

Blood samples were collected after an overnight fast at baseline, 3, 6, and 12 mo. The serum samples were aliquoted and stored at −80 °C for later analysis of several biomarkers. These included the C-terminal telopeptide of type I collagen (CTX), as a marker of bone resorption, procollagen I intact N-terminal peptide (P1NP), for bone formation, bone alkaline phosphatase (B-ALP), a marker of bone formation, 25OHD, and PTH. The measurements of CTX, P1NP, and B-ALP were performed using the IDS-ISYS Multi-Discipline Automated Analyzer based on chemiluminescence technology. The intra-assay coefficients of variation were 3.0% for P1NP and 2.0% for CTX. PTH was assessed by ELISA, with an intra-assay variability of 6% and an inter-assay variability of 7%. 25OHD was measured using the LIAISON 25OHD assay, with an intra-assay variability of 8% and an inter-assay variability of 12%. To reduce inter-assay variability, all samples were measured in a single batch. Other MM related markers were tested in the central laboratory of the University of Verona using standardized methods.

HR-pQCT was performed at baseline, month 6, and 12 with ARTiCAT RAR Srl with 65 μm voxel resolution at the distal radius of the nondominant hand. Given the presence of external material in the radius of one patient, the dominant hand was analyzed. The reference line on the positioning was 9.5 mm from joint space (radiocarpal). Bone microstructure was evaluated at the distal radius with the following parameters: trabecular volumetric BMD (Tb.vBMD, mgHA/cm^3^), total vBMD (Tot.vBMD, mgHA/cm^3^), cortical vBMD (Ct.vBMD, mgHA/cm^3^), trabecular bone volume fraction (Tb.BV/TV), trabecular thickness (Tb.Th, mm), trabecular separation (Tb.Sp, mm), and cortical thickness (Ct.Th, mm). Micro-finite element analysis (μFEA) was performed using proprietary software. Parameters of μFEA included estimated failure load (kN) and cortical stiffness (kN/mm).

### Sample size estimation

Sample size calculation was based on a one-sample, two-sided *t*-test comparing the mean percentage change in LS BMD to zero (detecting a significant increase from baseline to month 12). Using data from the ARCH trial,^27^ which reported a mean BMD increase of 13.7% (95% CI, 13.38-13.95) in the ROMO group (*n* = 2047), we estimated the standard deviation to be approximately 6.6%. This yields a standardized effect size (Cohen’s *d*) of about 2.08. To conservatively detect an effect size of |δ| ≥1.4 with 90% power and α = .05 (two-sided), a minimum of 8 participants is required.

### Statistical analysis

Descriptive statistics were presented as mean and standard deviations or median and interquartile range (IQR) as appropriate. We analyzed BMD, HR-pQCT parameters and serum markers changes over time with mixed-effect model analysis for repeated measures using Satterthwaite and restricted maximum likelihood method with time as fixed effect and patients as random effect. To account for multiplicity, we used the false discovery rate (FDR) approach with the two-stage step-up method of Benjamini, Krieger, and Yekutieli (*Q* value 5% of FDR). All differences were considered significant when *p* value was inferior to .05.

Statistical analyses were performed using SPSS Version 26 (SPSS, Inc.), GraphPad Prism version 9.5.1 (GraphPad Software), and JASP (Version 0.19.0). This study was approved by the University of Verona Ethic Committee (protocol registration: REUMABANK). All patients provided informed consent to participate in the study.

## Results

### Patient characteristics

**Figure 1:**
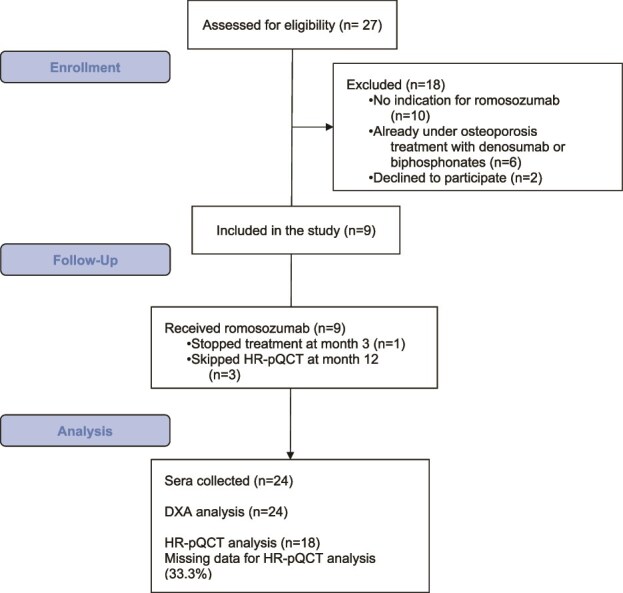
Study flowchart with the inclusion and exclusion criteria.

A total of 27 patients with a confirmed diagnosis of smoldering MM followed in the Hematology Department were consecutively assessed for fracture risk. Of these, only 9 were deemed suitable for ROMO treatment and satisfied enrollment criteria. One patient dropped out due to adverse event at month 3, and 8 patients concluded the treatment and evaluation. Figure 1 shows the patients’ disposition.

The demographic and clinical characteristics of the patients are depicted in Table 1. All patients had MM with immunoglobulin G (IgG) subtype, were female and had a mean age of 67.4 ± 9.9 yr old. None of the included patients received specific treatment for MM during the observation period. Five patients (62.5%) had a previous fragility fracture, and the mean BMD T-scores were −3.1 ± 1 at the LS, −1.5 ± 0.4 at the TH and −2 ± 0.8 at the FN. At baseline, median M-protein levels were 22.8 (18-27.3) g/L, free serum light chains ratio (K/λ) was 6.9 (0.3-40.8) and β2-microglobulin levels were 2.4 (2.1-3) mg/L.

**Table 1:** Baseline characteristics of the patients with MM that received romosozumab for 12 mo.

**Pt**	**Age**	**Sex**	**BMI** **(kg/m**^**2**^**)**	**Disease duration (years)**	**Smoking Status**	**Previous fracture**	**Lumbar Spine** **BMD (g/cm**^**2**^**) T-score**	**Femoral Neck** **BMD (g/cm**^**2**^**) T-score**	**Total Hip** **BMD (g/cm**^**2**^**)** **T-score**	**M-protein (g/L)**	**Serum FLC ratio (K/λ)**	**Fractures during FU**	**Disease progression during FU**	**After ROMO**
**1**	59	F	19.5	5	No	Yes (peripheric + vertebral)	0.956 −1.9	0.668 −2.6	0.782 −1.8	19.5 (λ)	0.5	No	No	ZOL
**2**	77	F	28.3	<1	No	Yes (vertebral)	0.810 −3	0.752 −1.9	0.833 −1.4	18 (λ)	0.1	No	No	DMAB
**3**	77	F	22.2	26	No	No	0.763 −3.4	0.695 −2.4	0.811 −1.6	18 (K)	34.5	No	No	DMAB
**4**	78	F	22.3	10	No	No	0.905 −3.2	0.826 −1.3	0.821 −1.5	14 (K)	101.5	No	No	DMAB
**5**	62	F	19.7	<1	No	No	0.550 −5	0.690 −2.4	0.736 −2.2	31 (K)	59.7	No	No	DMAB
**6**	51	F	21.8	10	Yes, actual	Yes (vertebral)	0.916 −2.2	0.798 −1.5	0.820 −1.5	28 (λ)	0.3	No	No	DMAB
**7**	71	F	30.9	1	Yes, actual	Yes (vertebral)	0.834 −2.9	0.981 −2	0.866 −1.1	26 (K)	13.3	No	No	DMAB
**8**	64	F	20.7	2	Yes, former	Yes (vertebral)	0.709 −3.8	0.745 −2	0.872 −1.1	27 (λ)	0.1	No	No	DMAB

Abbreviations: DMAB, denosumab; FLC, free light chains; FU, follow-up; ROMO, romosozumab; SMM, smoldering multiple myeloma; ZOL, zoledronic acid.

### Bone mineral density

**Figure 2:**
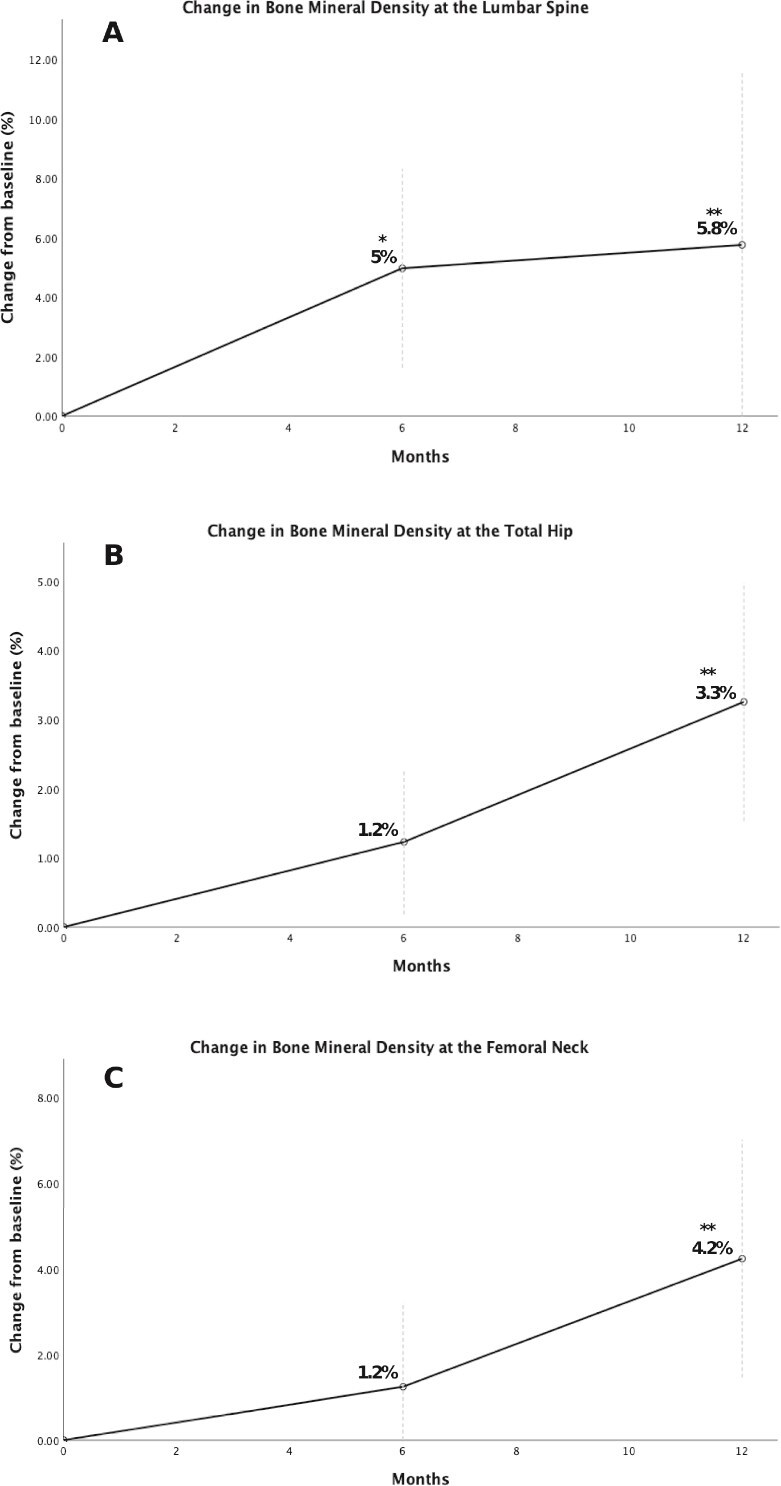
BMD percentage change in multiple myeloma patients treated with romosozumab. Panel A shows LS BMD, panel B shows TH BMD, and panel C shows FN BMD. ^*^p-value <.05 at 6 mo vs baseline. ^**^p-value <.05 at 12 mo vs baseline.

Over a period of 12 mo, there was a significant improvement in BMD in all evaluated sites (Table 2 and Figure 2). Lumbar spine BMD increased from median 0.822 (IQR 0.750-0.908) to 0.832 g/cm^2^ (IQR 0.752-0.908) at 12 mo (*p* = .046), corresponding to a percentage change of 5.8% at 12 mo (*p* = .048). At the FN, there was an increase from 0.749 (IQR 0.694-0.805) to 0.784 (IQR 0.729-0.836) g/cm^2^ at 12 mo (*p* = .015), corresponding to a 4.3% change (*p* = .020). At the TH, BMD improved from 0.821 (IQR 0.804-0.841) to 0.848 (IQR 0.260-0.884) g/cm^2^ (*p* = .003), with a percentage change of 3.3% at 12 mo (*p* = .002).

**Table 2:** Changes in laboratory and DXA parameters in multiple myeloma patients treated with romosozumab.

	**M0**	**M6**	**M12**	***p*-value**
**Laboratory parameters**				
**Hb (g/dL), median (IQR)**	12.5 (11.6-12.8)	12.2 (11.6-12.5)	12.2 (11.2-12.7)	.397
**RBC count (10** ^**3**^ **/μL), median (IQR)**	3.9 (3.6-4.1)	3.9 (3.7-3.9)	3.7 (3.5-4)	.293
**WBC/μL, median (IQR)**	5.8 (4.9-6.9)	5.8 (5.1-8.1)	5.8 (4.7-6.9)	.522
**Platelets (10** ^**3**^ **/μL), median (IQR)**	249.5 (223.8-331.8)	248.5 (237.3-278.5)	263.5 (241.5-304.0)	.320
**25 (OH) vitamin D (ng/mL), median (IQR)**	27.5 (26.0-45.9)	43 (33.0-44.6)	45.4 (35.9-56.1)	.090
**PTH (pmol/L), median (IQR)**	5.2 (4.1-5.5)	4.7 (4.3-5.3)	6.0 (4.1-6.4)	.468
**ALP (U/L), median (IQR)**	68.5 (61.3-73.3)	67.5 (57.8-79.3)	59 (52-66.5)	**.020**
**B-ALP (ng/mL), median (IQR)**	13.5 (9.3-17.0)	13 (10.5-15.0)	9.0 (6.0-12.0)	**.037**
**Ca (mg/dL), median (IQR)**	9.4 (9.3-9.7)	9.4 (9.1-9.5)	9.3 (9.2-9.6)	.495
**Pi (mg/dL), median (IQR)**	3.6 (3.4-3.7)	3.3 (3.1-3.6)	3.4 (3.1-3.9)	.282
**Cr (mg/dL), median (IQR)**	0.8 (0.6-0.9)	0.9 (0.6-1.0)	0.9 (0.6-1.0)	.122
**GFR (mL/min/1.73 m** ^**2**^ **), median (IQR)**	84.5 (65-93)	79.5 (62-94)	72.5 (59.3-92.8)	.324
**IgG (g/L), median (IQR)**	27.1 (23-31.8)	29.8 (24.1-33.3)	30.2 (26.2-33.2)	.460
**IgA (g/L), median (IQR)**	0.3 (0.3-0.5)	0.3 (0.3-0.5)	0.4 (0.3-0.6)	.307
**IgM (g/L), median (IQR)**	0.4 (0.3-0.4)	0.4 (0.3-0.5)	0.4 (0.3-0.5)	.518
**M-protein (g/L), median (IQR)**	22.8 (18-27.3)	22.5 (18.8-25.8)	23.3 (20.0-27.3)	.060
**Serum FLC: λ (mg/L), median (IQR)**	17.3 (7.6-43.3)	20.8 (9.1-75.4)	15.9 (10.1-47.5)	.895
**Serum FLC: K (mg/L), median (IQR)**	90.9 (10.9-239.4)	98.2 (10.5-249.6)	97.9 (9.8-411.8)	.181
**Serum FLC ratio (K/λ), median (IQR)**	6.9 (0.3-40.8)	8.1 (0.4-50.0)	8.1 (0.4-65.4)	.586
**Urine FLC λ/Cr (mg/mmol), median (IQR)**	1.5 (0-6.8)	3.0 (0-6.3)	3.0 (0-0.3)	.442
**Urine FLC K/Cr (mg/mmol), median (IQR)**	8.7 (0.1-30.5)	8.7 (0.1-21.5)	8.3 (0.1-19.8)	.256
**Total proteins (g/L), median (IQR)**	77.5 (76.3-87.2)	82 (78.8-84)	83.5 (79-86.5)	.337
**β2-microglobulin (mg/L), median (IQR)**	2.35 (2.1-3.0)	2.1 (1.8-2.8)	2.1 (1.8-2.5)	.092
**DXA parameters**				
**Lumbar spine BMD (g/cm** ^**2**^ **), median (IQR)**	0.822 (0.750-0.908)	0.850 (0.7.61-0.953)	0.832 (0.0.752-0.908)	**.046**
**Femoral neck BMD (g/cm** ^**2**^ **), median (IQR)**	0.749 (0.694-0.805)	0.759 (0.698-0.826)	0.784 (0.728-0.836)	**.015**
**Total hip BMD (g/cm** ^**2**^ **), median (IQR)**	0.821 (0.804-0.841)	0.835 (0.799-0.851)	0.848 (0.260-0.884)	**.003**

Abbreviations: ALP, alkaline phosphatase; B-ALP, bone alkaline phosphatase; Ca, calcium; Cr, creatinine; FLC, free light chains; GFR, glomerular filtration rate; Hb, hemoglobin; Ig, immunoglobulin; Pi, phosphorus; RBC, red blood cell; WBC, white blood cell.

### Bone turnover markers

**Figure 3:**
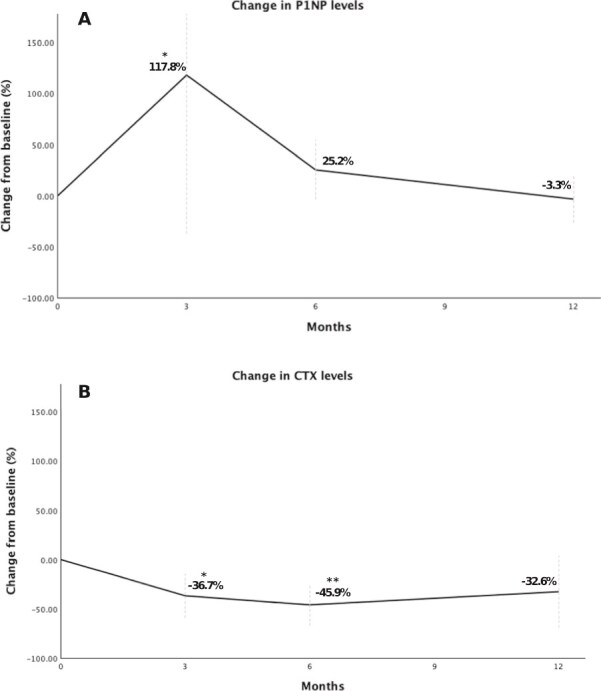
Bone turnover markers percentage change in multiple myeloma patients treated with romosozumab. Panel A shows P1NP changes and panel B shows CTX changes. ^*^p-value <.05 at 3 mo vs baseline. ^**^p-value <.05 at 6 mo vs baseline.

Treatment with ROMO led to significant changes in several BTMs. Serum alkaline phosphatase (ALP) and B-ALP showed a significant decrease by month 12: 68.5 (IQR 61.3-73.3) to 59 (IQR 52.0-66.5) U/L (*p* = .020) and 13.5 (IQR 9.3-17.0) to 9 (IQR 6.0-12.0) ng/mL (*p* = .022), respectively. P1NP rose sharply by month 3, from 60 (49.5-70.3) to 98 (77-115.5) ng/mL (mean percentage change of 117.8%, Figure 3), but then declined, approaching baseline by month 12 (*p* = .006). Conversely, CTX showed a tendency for a reduction at follow-up points (*p* = .060), with a maximal decrease at the end of the 12 mo (−53.6%, Figure 3).

### Multiple myeloma activity markers

No significant changes were observed in serum immunoglobulins (IgG, IgA, and IgM), M-protein levels or serum, and urine FLCs, suggesting overall stable disease activity over the study period (Table 2). Serum β2-microglobulin decreased at M12 from baseline (from 2.35 to 2.1 mg/L, *p* = .042). However, when considering the entire trajectory across all time points (baseline, M6, and M12), the overall trend was not significant (*p* = .092).

### High-resolution peripheral quantitative computed tomography evaluation

Volumetric BMD (Tot.vBMD, Ct.vBMD, and Tb.vBMD) at baseline, 6 mo, and 12 mo did not change significantly. However, there was an increase in estimated failure load at 6 mo from 1.06 (0.99-1.16) to 1.35 (1.27-1.51) kN, *p* = .033, that was not sustained through month 12 (*p* = .079). Additionally, no changes were observed in other parameters, with a stable evolution of Ct.Th, Tb.BV/TV, Tb.Th, Tb.Sp, and HR-pQCT parameters changes are reported in Table 3.

**Table 3:** HR-pQCT at distal radius in in multiple myeloma patients treated with romosozumab.

	M0	M6	M12	*p*-value
**Tot.vBMD (mgHA/cm^3^)**	303.82 (285.59-363.79)	308.83 (295.00-365.39)	301.33 (282.78-325.48)	.987
**Ct.vBMD (mgHA/cm^3^)**	740.12 (738.29-840.76)	745.57 (740.86-854.81)	771.05 (720.30-800.41)	.610
**Tb.vBMD (mgHA/cm^3^)**	162.34 (148.86-204.24)	170.30 (165.59-183.59)	177.91 (152.37-196.99)	.821
**Ct.Th (mm)**	0.73 (0.71-0.76)	0.76 (0.71-077)	0.73 (0.69-0.75)	.399
**Tb.BV/TV (%)**	12.07 (11.65-12.64)	12.53 (11.88-13.01)	12.65 (11.24-13.74)	.832
**Tb.Th (mm)**	0.28 (0.27-0.28)	0.27 (0.26-0.28)	0.27 (0.27-0.27)	.603
**Tb.Sp (mm)**	0.82 (0.82-0.84)	0.81 (0.81-0.83)	0.83 (0.78-088)	.910
**Stiffness (kN/mm)**	36.88 (36.70-37.46)	37.19 (36.62-37.25)	36.62 (35.16-36.94)	.215
**Failure load (kN)**	1.06 (0.99-1.16)	1.35 (1.27-1.51)	1.16 (1.06-1.27)	.079

Abbreviations: Ct.vBMD, cortical vBMD; Ct.Th, cortical thickness; Tot.vBMD, total volumetric BMD (vBMD); Tb.BV/TV, trabecular bone volume fraction; Tb.Sp, trabecular separation; Tb.vBMD, trabecular vBMD.

### Fractures and skeletal related events to multiple myeloma

No clinical fractures were reported during the follow-up. No new radiographic vertebral fractures were observed with VFA at follow-up. None of the patients had clinical progression of the underlying disease.

### Safety

No adverse events, including osteonecrosis of the jaw, atypical femoral fractures, or serious cardiovascular adverse events, were registered during the study period. One patient reported arthralgias and myalgias during the first months of treatment, and ROMO was discontinued at month 3.

## Discussion

In this study, we provide novel evidence that ROMO may improve bone health in patients with MM, without adversely affecting the underlying disease.

Osteoporosis is common in MM but is neither included in its diagnostic criteria nor classified as a SRE.^4^ Multiple myeloma patients have higher fracture risk compared to the general population, regardless of disease stage.^9^^,^^29^ Currently recommended treatments in patients with MM and osteoporosis are limited to bisphosphonates and denosumab, but high-risk patients might benefit from broader therapeutic strategies.^26^

Herein, we showed a significant increase in BMD at LS, FN, and TH during the 12 mo of treatment with ROMO in MM. While these gains align with findings from pivotal osteoporosis trials, the magnitude of increase in BMD in our cohort was lower, especially at the LS.^27^^,^^30^ This attenuated response may reflect the differences in bone microenvironment in MM patients, particularly in trabecular bone, which could limit the full potential of ROMO.

Bone turnover markers inform on important bone dynamics and, particularly in MM, can reflect disease activity.^31^ The changes in BTMs were again in line with previous studies in postmenopausal women without MM.^27^^,^^30^ P1NP, gold-standard marker of bone formation, peaked after 3 mo of treatment and then returned within the baseline range. In contrast, CTX marker of bone resorption and B-ALP declined progressively throughout the follow-up.

Importantly, MM did not progress throughout the study period. No significant changes in M-protein, immunoglobulin levels, or FLCs were observed. While serum β2-microglobulin showed a modest decline over time, this was not sustained across all time points. β2-microglobulin is a well-known prognosis marker in MM, and its levels associate with more aggressive disease and poorer survival.^32^ The small effect observed in our study may reflect biological variability rather than disease-modifying effect. However, these results are reassuring and demonstrate that ROMO did not appear to promote MM progression.

Monitoring treatment response with HR-pQCT allowed for a close analysis of both trabecular and cortical architectural parameters and volumetric BMD and may contribute to a better understanding of the treatment-related effects on cortical and trabecular compartments, that is not captured by DXA or biochemical analysis.^33^ Indeed, patients with early-stage MM have lower volumetric BMD, microarchitecture defects and lower cortical stiffness, compared to controls.^34^

In our study, ROMO led to a transient increase in failure load at 6 mo, which was not sustained at 12 mo. The observed increase in failure load may reflect small microarchitectural improvements, suggesting enhanced bone strength with ROMO, particularly at the 6-mo timepoint. Although no changes in volumetric BMD and other microarchitectural parameters were observed, the absence of deterioration suggests that ROMO preserves bone structural integrity even in MM patients, who are prone to accelerated bone loss.

Our study has several limitations. Though we were able to detect significant differences in BMD and BTMs with ROMO treatment, the small sample size may have precluded our ability to detect significant differences in other biochemical and HR-pQCT parameters. All patients in our study received daily vitamin D supplementation, which could have contributed to the observed improvements in bone parameters. However, all randomized controlled trial of ROMO that inform current guidelines included vitamin D supplementation and this is in accordance with standard clinical practice. Additionally, the absence of an active comparator limits the interpretation of the results. This study was conducted in MM patients without CRAB, so the generalization of our findings to patients with more advanced disease is not possible. Nonetheless, these results provide preliminary insight into the potential role of ROMO in MM patients with osteoporosis and highlight the need for further investigation.

In conclusion, patients with MM may benefit from ROMO treatment—even in the absence of CRAB—to mitigate skeletal fragility and preserve bone microarchitecture. Further studies with larger samples and an adequate control group may better elucidate the role of ROMO in patients with active MM regarding bone healing and myeloma progression.

## Ethics approval and consent to participate

The study was conducted according to the protocol REUMABANK approved by our local Ethics Committee, in accordance with the 1964 Helsinki declaration and its later amendments or comparable ethical standards. Informed consent was collected for each participant.

## Consent for publication

Patients provided consent for publication of anonymized data.

## Data Availability

Data of the analysis is available upon reasonable request.
